# Impact of Age and Comorbidities On Therapeutic Decision-making Among Older Patients With Gastrointestinal Cancer

**DOI:** 10.1007/s12029-025-01337-2

**Published:** 2025-10-27

**Authors:** Marianna Javier-González, Raffaele Galli, Carlos Amorós Rivera, Robert Rosenberg, Marcus Vetter

**Affiliations:** 1 Department of Oncology and Hematology, Cantonal Hospital Baselland, Liestal, Switzerland; 2 Department of General and Visceral Surgery, Cantonal Hospital Baselland, Liestal, Switzerland; 3 Faculty of Medicine, University of Valladolid, Valladolid, Spain; 4 Faculty of Medicine, University of Basel, Basel, Switzerland

**Keywords:** Age, Comorbidity, Gastrointestinal cancers, Geriatric assessment, Treatment guidelines

## Abstract

**Purpose:**

The impact of age, comorbidities and geriatric syndromes is often overlooked during tumor board (TB) decisions. We investigated how frequently age, comorbidities, functional and nutritional parameters, and frailty are mentioned when deciding treatments for older adults with gastrointestinal (GI) cancers, and the impact these variables have on adherence to TB decisions and treatment guidelines from the European Society for Medical Oncology (ESMO).

**Methods:**

Cross-sectional study of data from patients aged ≥ 65 years presented before the GI-TB of a tertiary cancer center between July 2019 and December 2022 based on electronic health records and TB documentation. Mention of age, comorbidity, functional and nutritional parameters, and frailty at decision-making, and adherence to TB decisions and treatment guidelines were assessed.

**Results:**

418 patients with a mean age of 77 years and Charlson Comorbidity Index (CCI) of 8.6 were included. Geriatric variables were mentioned in 43.8% of cases. Among these, comorbidities were mentioned in 17.2%, whereas age was mentioned in 14.6%. Adherence to TB decisions was 82%, whereas adherence to ESMO guidelines was 69%. Mention of age and comorbidity was associated with a 2-fold and 3-fold reduction in the likelihood of adherence to ESMO guidelines (*p* = 0.02 and 0.001, respectively). This association was not found when analysing adherence to TB decisions.

**Conclusions:**

Geriatric variables, despite being often neglected at the time of defining treatment for the older adult with cancer, can have an effect on oncologic decision-making. Our findings underscore the need for integrating assessment of geriatric variables into oncologic care to support individualized, guideline-concordant treatment planning that reflects the complexity and needs of this population.

## Key Summary Points

**Aim:**

Cross-sectional study to investigate how often age, comorbidities, functional and nutritional parameters, and frailty are taken into account at the time of oncologic treatment decision making.

**Findings:**

Geriatric variables, predominantly comorbidity, were mentioned in 43.8% of cases during TB review. Mentions of age and comorbidities were associated with 2–3-fold lower adherence to ESMO guidelines.

**Message:**

The impact of age, multiple comorbidities, and geriatric syndromes is often overlooked during treatment decision-making.

## Introduction

Gastrointestinal (GI) cancers represent more than 25% of the global cancer incidence [1], and more than two-thirds of people with GI cancer are older than 65 years [2]. Older persons with cancer present with considerable heterogeneity due to multimorbidity, polypharmacy, and diverse functional and cognitive statuses that often complicate treatment decision-making for clinicians. Given the high incidence of cancer among older adults, it is crucial that healthcare providers understand and integrate the unique needs of this group into oncologic care.

Oncologic decision-making is inherently complex, requiring the integration of evidence on survival, tumor control, adverse effects, and costs, often guided by standardized protocols [3, 4] that may incorporate age. Age-related factors, such as comorbidities, polypharmacy, frailty, and functional decline, are strongly associated with poorer clinical and oncologic outcomes, including treatment toxicity, survival, and hospital readmission [5–10]. These factors also contribute to the frequent exclusion of older adults from clinical trials, leading to a lack of representative data for this population [11–14]. In particular, trials targeting older adults with GI cancer are scarce and rarely incorporate a Comprehensive Geriatric Assessment (CGA) or patient-centered outcomes [15]. 

Based on these observations and the heterogeneity of the older population with GI cancer, individualized approaches and shared decision-making in the framework of a multidisciplinary team are needed [16]. International societies have published recommendations for treating people older than 65 years with cancer to help improve outcomes and avoid over- and undertreatment in these patients [17–19]. Treatment plans based on a holistic evaluation of function, cognition, nutrition, frailty, and other variables have been associated with improved survival, reduced complications, and better quality of life in older cancer patients [20–22]. At the same time, measures of function, nutrition, and mental health are important predictors of 1-year mortality in older adults with GI cancers [23]. 

Despite this growing body of evidence, a geriatric assessment (GA) and GA-driven management (GAM) are not universally offered to older people with GI cancer in usual oncologic practice worldwide. Skepticism regarding its added value and barriers affecting implementation, including a perception of increased clinic time and burdensome assessments [24], resource availability, professional dynamics, and patient-related factors [25], hinders the systematic application of a GA and collaborative geriatric-oncology models. As a result, treatment planning for older adults with cancer often follows a single-disease model that overlooks the multifaceted needs of this population.

There is limited evidence on how geriatric-specific variables are incorporated into real-world oncologic decision-making for older adults with GI cancer, particularly within Tumor Board (TB) discussions. Little is known about whether and how factors such as functional and nutritional status, and frailty are weighed alongside traditional oncologic indicators, or how they influence therapeutic recommendations.

The present study aimed to investigate the extent to which geriatric variables, including age, comorbidities, functional and nutritional status, and frailty criteria, are considered during TB discussions in a Swiss tertiary cancer center. We also assessed the impact of these variables on patient prognosis and adherence to both internal TB and international treatment guidelines.

This study reflects current practice in a real-world setting and aims to support the integration of geriatric principles into oncology, advocating for individualised, patient-centred treatment decisions for older adults with GI cancers.

## Methods

### Design

We retrospectively collected cross-sectional data of patients 65 years and older presented before the TB for GI cancers in a Swiss tertiary cancer center between July 2019 and December 2022. Since July 2019, the TB has been divided according to specialty, resulting in a dedicated TB for GI cancers. Before this date, all tumors were presented at a single weekly meeting. Members of the TB include medical and radiation oncologists, gastrointestinal surgeons, gastroenterologists, abdominal radiologists, psycho-oncologists, pathologists, palliative care specialists, and specialists from other disciplines, depending on the individual case, such as urologists, gynaecologists, and vascular surgeons.

After presentation before the TB, a decision summary containing personal data, diagnosis, medical history, medication, complementary tests, including pathology, psychological distress scales, and therapeutic recommendations, is written for each patient. These summaries are filed in the electronic health records (e-HR).

### Study Cohort

All patients 65 years and older with a new diagnosis of GI cancer or with recurrences of a known GI cancer presented before the TB for GI cancers were included. A threshold of 65 years was used to define the older adult population, consistent with commonly used definitions in epidemiological studies and previous oncology research. Subgroup analyses were also conducted across finer age strata (65–69, 70–79, 80–89, and ≥ 90 years) to capture heterogeneity within the older population.

We excluded patients without a signed general informed consent form (ICF) for the use of health-related data for research purposes and patients whose data were hidden in the e-HR because of privacy concerns. Patients who were found to have a non-GI origin of the primary tumor, a non-malignant condition, or neuroendocrine neoplasms (NEN) of the GI tract were also excluded.

A non-probabilistic, consecutive sampling technique was used. Nevertheless, taking into account the yearly incidence of cancer in our region, a sample of 382 patients was considered to be representative of our population.

### Data Collection

Records were retrospectively reviewed, and demographic and healthcare data were collected and recorded in a database designed for this study. The Research Electronic Data Capture (REDCap) Software hosted by the Cantonal Hospital of Baselland was used for data collection and management [26, 27]. 

The e-HR were further reviewed to collect information on the place of residence, treatment received, and adverse outcomes, including unplanned hospitalizations and mortality, by the time of data collection.

Data on mortality were collected from the regional civil register when not available in the e-HR.

## Data Variables

Age, comorbidity, functional and nutritional parameters, and criteria used to describe frailty were defined as “geriatric variables”.

Content analysis of the TB summaries was performed and variables such as age, sex, tumor type and staging, comorbidities, type of treatment proposed, and mention of geriatric variables were extracted.

Observations used to justify proposed treatment, such as “due to age”, “due to comorbidities”, documentation of functional parameters, including the Functional Independence Measure score, Barthel Index, Performance Status (PS), or nutritional parameters, such as body mass index or the Nutritional Risk Score, as well as the use of the word “cachexia” or “low weight”, and documentation of frailty criteria, including the Clinical Frailty Scale, Geriatric-8 score, Fried’s criteria, or use of the words “weakness” or “fatigue”, were recorded.

Therapeutic recommendations were categorized and recorded as curative, palliative, or Best Supportive Care, the former two including surgery, chemotherapy, radiotherapy, or any combination.

Mortality was measured from the date of presentation before the TB for GI cancer, and the date of death recorded at data collection.

Comorbidities were extracted from medical records and coded using the Charlson Comorbidity Index (CCI) [28]. The Union for International Cancer Control (UICC) system was used to define tumor staging.

Adherence to TB decisions and ESMO guidelines was defined as consistency between the therapeutic recommendations documented in the TB summary and the treatment received. Treatment algorithms of the ESMO Clinical Practice Guidelines for Gastrointestinal Cancers [29] were reviewed to determine adherence.

### Statistical Analysis

Descriptive statistics were used to summarize patient demographics, tumor type and stage, mention of geriatric variables, and unplanned hospital visits. These data were extracted using the REDCap Software and are presented as quantitative measures. Normally distributed variables such as age and CCI are reported as means and standard deviations (SD). Given its distribution, mortality is presented using both mean and median values.

Statistical comparison of categorical variables (e.g. mention of geriatric variables, adherence to TB decisions, and ESMO guideline adherence) was conducted using Fisher’s exact test. As age and CCI were not normally distributed, group comparisons were performed using the Mann–Whitney U test.

Multivariate analysis was performed using binary logistic regression to assess whether the mention of geriatric variables predicted adherence to TB decisions and ESMO guidelines. Results are reported as odds ratio (OR) with corresponding confidence intervals (CI). The model was adjusted for age at presentation, CCI excluding age, and CCI including tumor (with or without metastasis).

Survival analysis was conducted using Kaplan–Meier estimates to compare outcomes between patients in whom age and comorbidities were mentioned during treatment decision-making versus those in whom they were not.

All analyses were performed using SPSS version 15.0 (IBM SPSS Inc., Armonk, NY, USA). A p-value < 0.05 was considered statistically significant.

### Ethics Approval

Since November 2018, all patients admitted to our institution or their legal guardians are invited to sign a general consent for the collection, storage, and use of healthcare data for education and research. The ICFs are stored in the e-HR.

This project has been approved by the Ethics Committee of Northwest Switzerland with the number 2023 − 00996.

## Results

### Participant Selection

**Fig. 1:**
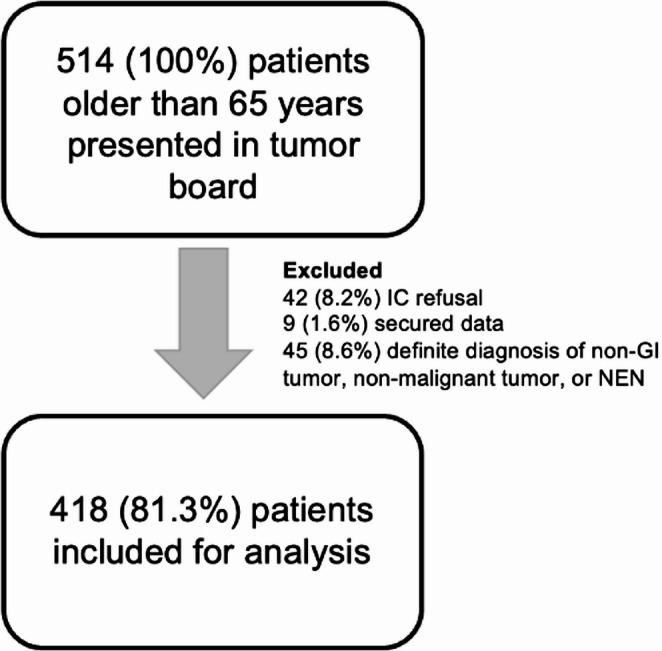
Inclusion process. IC, Informed Consent; NEN, Neuroendocrine Neoplasm

We identified 514 patients with a suspected cancer diagnosis who were eligible for inclusion. Of these, 42 were excluded for declining to sign the ICF, and nine were excluded due to protected data in the e-HR. After inclusion, 45 patients were excluded due to a confirmed diagnosis of a benign condition, a NEN, or a non-GI origin of the primary tumor. A total of 418 patients aged 65 years and older with a diagnosis of GI tumor, including esophageal, gastric, small bowel, colon, rectum, anal, hepatobiliary, and pancreatic cancers, were included in the final analysis. The inclusion process is depicted in Fig. 1.

### Participants Characteristics

As shown in Table 1, the mean age of participants was 77 years (SD 7.36). The majority were male (59.3%), lived at home (93.1%), and were most commonly diagnosed with colon cancer (35.2%). Stage IV disease was the most frequent stage at inclusion (34.2%). The median CCI was 8.6 points (range 4–19), with congestive heart failure (30.1%) being the most prevalent comorbidity.

**Table 1:** Baseline characteristics of patients aged ≥ 65 years with Gastrointestinal cancers (*n* = 418)

Characteristic	*n* (%) or Mean ± SD (Range)
Age (years)	77 ± 7.36 (65–97)
Sex	
Female	170 (40.7)
Male	248 (59.3)
Place of living	
Home	389 (93.1)
Nursing home	29 (6.9)
Tumor site	
Colon	147 (35.2)
Rectum	72 (17.2)
Pancreas	62 (14.8)
Hepatobiliary	53 (12.7)
Gastric	41 (9.8)
Esophagus	34 (8.1)
Anal	7 (1.7)
Small bowel	2 (0.5)
Tumor stage (UICC)	
I	71 (16.9)
II	91 (21.8)
III	82 (19.6)
IV	143 (34.2)
Not staged	31 (7.4)
Comorbidities	
CCI (points)	8.6 (4–19)
Congestive heart failure	126 (30.1)
Myocardial infarction	67 (16.0)
Diabetes	81 (19.4)
COPD	54 (12.9)
Renal disease	42 (10.0)
Liver disease	40 (9.6)
Cerebrovascular disease	42 (10.0)
Dementia	34 (8.1)

CCI, Charlson Comorbidity Index; SD, standard deviation; UICC, Union for International Cancer Control

### Use of Geriatric Variables

At least one geriatric variable was mentioned during the TB review in 43.8% of cases. Subjective descriptors were most frequently used. The term “comorbidities” was the most commonly mentioned variable (17.2%), followed by “age” (14.6%), functional parameters (13.6%), and nutritional parameters (12.9%). Of the nutritional descriptors, subjective findings such as “low weight” and “cachexia” were referenced in 75.9% of cases. Frailty was mentioned in only 6.5% of patients. Table 2 summarizes the use of geriatric variables during TB discussion.

**Table 2:** Geriatric variables mentioned during tumor board discussions in patients aged ≥ 65 years with Gastrointestinal cancers

Variable category	*n* (%)	Subcategory	*n* (% within category*)
Any geriatric variable	183 (43.8)	—	—
Age	61 (14.6)	—	—
Comorbidity	72 (17.2)	—	—
Functional parameters	57 (13.6)		
		Subjective	52 (91.2)
		ECOG-PS	4 (7.0)
		Karnofsky PS	1 (1.8)
		Barthel Index	1 (1.8)
Nutritional parameters	54 (12.9)		
		Subjective	41 (75.9)
		BMI	4 (7.4)
		NRS	11 (20.4)
Frailty	27 (6.5)		
		Subjective	22 (81.5)
		G8	3 (11.1)
		CFS	2 (7.4)

*Percentages may exceed 100% as multiple measures could apply per patient

BMI, body mass index; CFS, Clinical Frailty Scale; ECOG-PS, Eastern Cooperative Oncology Group Performance Status; G8, Geriatric 8-score; NRS, Nutritional Risk Score

### Adherence to TB Decisions

Among patients with available treatment data (*n* = 398; 95.2%), 82% (*n* = 327) received treatment aligned with the TB recommendation. In the remaining 18% (*n* = 71), reasons for deviation included patient’s decision (*n* = 42; 60%), clinical deterioration or complications (*n* = 31; 44%), and death before initiation of therapy (*n* = 4; 6%).

### Adherence to ESMO Guidelines

Of the 350 patients (84.7%) with available treatment data for this analysis, 69% (*n* = 242) received care consistent with ESMO guidelines, while 31% (*n* = 108) did not. The most common reasons for non-adherence were personal choice, clinical situation, and advanced renal disease.

### Impact of Mentioning Geriatric Variables on Oncologic decision-making

When analyzed individually, none of the geriatric variables were significantly associated with adherence to TB decisions (Table 3). However, in subgroup analysis by age group, the mention of comorbidities was associated with a reduced likelihood of adherence to TB decisions in the 65–69 and 80–89 age groups (*p* = 0.033 and *p* = 0.029, respectively).

**Table 3:** Association between geriatric variables and adherence to tumor board recommendations (*n* = 398*)

Variable mentioned	OR	95% CI	*p*-value
Age	1.19	0.59–2.37	0.63
Comorbidities	0.53	0.24–1.17	0.12
Functional parameters	0.71	0.32–1.56	0.39
Nutritional parameters	1.06	0.51–2.23	0.88
Frailty	1.14	0.41–3.15	0.80

*n = 398. Missing data for 20 patients (4.8%). Adherence to tumor board recommendation observed in 327 of 398 patients (82.2%)

OR, odds ratio; CI, confidence interval

Regarding adherence to ESMO guidelines, the mention of age or comorbidities during decision-making was associated with a significantly reduced likelihood of receiving guideline-consistent care (OR 3.2 and 3.8, respectively; *p* < 0.001) (Table 4). These associations remained significant in multivariate analysis, with a two-fold reduced likelihood of receiving care according to ESMO guidelines if age was mentioned (95% CI: 1.11–4.07, *p* = 0.02), and a three-fold reduction when comorbidities were mentioned (95% CI: 1.64–5.64, *p* = 0.001).

**Table 4:** Association between geriatric variables and adherence to ESMO guidelines (*n* = 350*)

Variable mentioned	OR	95% CI	*p*-value
Age	3.19	1.76–5.77	< 0.001
Comorbidities	3.82	2.18–6.70	< 0.001
Functional parameters	1.06	0.55–2.05	0.87
Nutritional parameters	1.28	0.65–2.53	0.47
Frailty	1.13	0.44–2.88	0.80

*N = 350. Missing data for 68 patients (16.3%). Adherence to ESMO guidelines observed in 242 of 350 patients (69.1%)

OR, odds ratio; CI, confidence interval; ESMO, European Society for Medical Oncology

Subgroup analysis by age group revealed no significant association between mention of age and guideline adherence, likely due to small sample sizes in the 65–69 and 90–99 age groups. In contrast, the mention of comorbidities maintained a significant inverse association with guideline adherence in the 70–79 age group.

No significant associations were found between the mention of functional, nutritional parameters, or frailty parameters and adherence to ESMO guidelines, potentially due to the low frequency of use.

An inverse association was found between higher age and CCI and adherence to ESMO guidelines (Mann-Whitney test: Z= −5.88 and Z= −4,4, *p* < 0.001, respectively). This association remained significant when CCI was calculated excluding age (Z= −2.96, *p* = 0.003).

No such association was observed regarding adherence to TB decisions.

### Survival and Mention of Geriatric Variables

Patients for whom comorbidities were mentioned during decision-making showed a non-significant trend towards longer survival compared to those without such mention (206 vs. 130 days, *p* = 0.074). No significant difference in survival was observed based on whether age was mentioned (174 vs. 133 days, *p* = 0.391).

### Survival Analysis

Survival data were available for 339 patients (98.1%), of whom 211 (62%) had died by the time of data collection. The mean survival was 224 days (SD 16.5), and the median survival was 140 days.

### Adverse Events

A total of 240 patients (57.4%) had at least one unplanned hospital visit, including Emergency Department attendances or hospitalizations. The most common reasons were cancer-related complications, followed by exacerbation of chronic conditions or unrelated acute events.

## Discussion

This study evaluated the frequency and influence of incorporating geriatric variables into therapeutic decision-making by a TB in older adults with GI cancers. We found that these variables, particularly comorbidity, were mentioned in less than half of TB discussions, and their mention was associated with a reduced likelihood of adherence to ESMO treatment guidelines.

Despite a mean age of 77 years in our cohort, age was explicitly mentioned in only 14.6% of cases. This could suggest a conscious effort by the multidisciplinary team to avoid age-based decision-making and promote treatment equity. However, the omission of age-related considerations may also pose risks, as biological aging brings physiological changes that can alter treatment tolerance and toxicity profiles [30]. Older patients with cancer often experience conditions such as sarcopenia, malnutrition, cognitive impairment, and polypharmacy, which are independently associated with adverse outcomes [31–33]. Ignoring these factors may lead to both under- and overtreatment. A recent study by Goggin et al. similarly found age-related variability in treatment decisions for older adults with GI cancers, underscoring the importance of structured geriatric assessment (GA) to guide individualized care [34]. 

Our findings align with previous studies in other tumor types. Ring et al. showed that age and comorbidities were among the most influential factors affecting oncologic treatment decisions, with comorbidities often exerting a greater influence than chronological age [35, 36]. This reflects increasing recognition that comorbid conditions may better predict clinical outcomes and treatment risks. In our cohort, comorbidities were mentioned in 17.2% of cases, but structured tools such as the Charlson Comorbidity Index (CCI) were not formally integrated into TB discussions.

Functional status, a well-established predictor of survival in older cancer patients, including those with colorectal cancer [37–39], was mentioned in only 13.6% of cases in our study. This represents a missed opportunity to tailor treatments to individual patients’ needs. Similarly, nutritional parameters were referenced in 12.9% of discussions, and just 27.8% of those cases were based on objective measures. Given that malnutrition is highly prevalent among older adults with GI cancer, and that guidelines from societies such as Enhanced Recovery After Surgery (ERAS) Society and the European Society for Clinical Nutrition and Metabolism (ESPEN) recommend routine nutritional screening [40], this low rate raises concerns. One possible explanation is that nutritional assessments are conducted but not adequately documented or communicated within the TB context. Regardless, this gap warrants internal review and quality improvement efforts.

Frailty, a clinical state of vulnerability to adverse outcomes [41], was mentioned in just 6.5% of cases, mostly based on subjective impressions. Only five patients had frailty assessed using a validated tool. Considering that up to 40% of older adults with GI cancers are frail and that frailty is associated with poor treatment tolerance and increased mortality [8, 42] this low rate highlights a lack of awareness or integration of geriatric syndromes in routine oncologic decision-making. In an international survey by Banna et al., fewer than 50% of oncology professionals reported using any formal frailty screening, largely due to time constraints and limited familiarity with available tools [43]. 

Notably, the mention of age and comorbidities during TB discussions was associated with a significantly reduced likelihood of receiving ESMO guideline-adherent treatment. This is likely multifactorial, reflecting both clinical judgment about treatment suitability and patient preferences. While deviation from guidelines is not inherently inappropriate, especially in complex older adults, it raises important questions about whether current guidelines sufficiently accommodate geriatric complexity. A systematic review found that non-adherence to oncology guidelines ranged from 8.2% to 65.3%, with most cases involving intentional deviation due to contraindications or patient-specific factors [44]. Similarly, Mutasingwa et al. found that only a third of clinical guidelines adequately addressed comorbidities in older patients [45]. Despite efforts by some organizations, such as the National Comprehensive Cancer Network (NCCN), most oncology guidelines still target a relatively healthy, younger population [46]. 

Our findings on age and CCI further reinforce this issue: higher age and comorbidity burden were both independently associated with lower adherence to ESMO guidelines. These results are in line with studies in breast and lung cancer populations, where increasing age and higher CCI scores predicted reduced guideline-concordant care [47, 48]. A vignette-based study by Büttelmann et al. found significant variability in treatment recommendations for older patients with comorbidities, suggesting inconsistency in applying standard protocols. Interestingly, even when GA results were provided, they did not reduce variability in recommendations, highlighting a potential disconnect between geriatric data and clinical application [49]. 

Survival data in our study offer further context. More than 50% of our patients had died by the time of data collection, with a median survival of 140 days. This compels reflection about the distribution of resources, as well as aiding honest conversations with patients and their relatives, establishing goals of care in line with patients’ preferences and values. Recent studies have found that older adults with cancer will prioritize outcomes such as function, cognition, and quality of life over survival and progression-free survival [50–52]. Exploring these preferences should become an integral part of defining treatment strategies.

Interestingly, we observed a non-significant trend toward longer survival among patients whose comorbidities were explicitly mentioned during TB discussions. Although this finding did not reach statistical significance, it may suggest that greater attention to geriatric factors leads to more personalized and appropriate care. Further research is needed to confirm this possible association.

To our knowledge, this is the first study to explore the impact of age and comorbidity on adherence to both internal TB decisions and international oncology guidelines in older adults with GI cancers. The large cohort, real-world setting, and inclusion of multiple GI tumor types enhance the generalizability of our findings. Importantly, our results highlight current gaps in integrating geriatric principles into oncologic care and suggest specific areas for improvement.

A geriatric-oncology collaboration has recently been established at our center, including the implementation of CGA for selected older patients presented at TB meetings. Future analysis of this new cohort, in comparison with our current findings, could provide valuable insights into the effect of GA-informed decision-making on adherence to guidelines and patient outcomes.

### Limitations

A few limitations should be acknowledged. First, all data were extracted from TB forms and e-HR, which may not fully reflect the scope of discussions held during TB meetings. While it is expected that key variables influencing decisions are documented, informal or verbal considerations of geriatric variables may have occurred without being recorded. Therefore, the frequency of geriatric variable usage may be underestimated.

Second, due to the low documentation rate for functional, nutritional, and frailty, we were unable to robustly assess the influence of these factors on treatment adherence or survival.

Third, patients were presented at different points in their disease trajectory, some at diagnosis, others at progression or complication, which could influence treatment decisions and the applicability of ESMO guidelines. This heterogeneity may have introduced variability not fully accounted for in the analysis.

Survival data were collected at a single time point, and follow-up duration varied between patients. This limits the precision of survival estimate and may introduce bias.

Finally, this study presents some potential selection bias. The study was conducted in a single cancer center in Switzerland, which may limit generalizability. Patients presented to the internal TB may differ systematically from those treated in community settings or not referred to TB at all.

Despite these limitations, the study provides valuable real-world insight into current practices and highlights the need for systematic integration of geriatric principles into oncologic decision-making. These findings can serve as a baseline for evaluating the impact of future interventions, such as the implementation of CGA in TB workflows.

## Conclusions

This study highlights that geriatric variables are frequently underrecognized in therapeutic decision-making for older adults with gastrointestinal cancers. Our findings suggest that age and comorbidities, when considered, can significantly influence treatment planning and guideline adherence, yet key factors such as functional status, nutrition, and frailty remain largely undocumented. Improving cancer care for older adults requires systematic acknowledgment of their clinical complexity, ideally through the routine use of standardized tools such as the CGA. Integrating geriatric principles into oncologic practice is essential to support individualized, evidence-based treatment strategies that align with the needs, capacities, and preferences of this growing patient population.

## Data Availability

The data that support the findings of this study are available upon request in the institutional REDCap database of the Cantonal Hospital of Baselland.
